# Predictability of machine learning techniques to forecast the trends of market index prices: Hypothesis testing for the Korean stock markets

**DOI:** 10.1371/journal.pone.0188107

**Published:** 2017-11-14

**Authors:** Sujin Pyo, Jaewook Lee, Mincheol Cha, Huisu Jang

**Affiliations:** Department of Industrial Engineering, Seoul National University, Seoul, South Korea; Utah State University, UNITED STATES

## Abstract

The prediction of the trends of stocks and index prices is one of the important issues to market participants. Investors have set trading or fiscal strategies based on the trends, and considerable research in various academic fields has been studied to forecast financial markets. This study predicts the trends of the Korea Composite Stock Price Index 200 (KOSPI 200) prices using nonparametric machine learning models: artificial neural network, support vector machines with polynomial and radial basis function kernels. In addition, this study states controversial issues and tests hypotheses about the issues. Accordingly, our results are inconsistent with those of the precedent research, which are generally considered to have high prediction performance. Moreover, Google Trends proved that they are not effective factors in predicting the KOSPI 200 index prices in our frameworks. Furthermore, the ensemble methods did not improve the accuracy of the prediction.

## Introduction

Predicting the trends of financial markets is one of the most important tasks for investors. They have tried to predict the trends using various methods and bet in the markets. Technical analysis and fundamental analysis are employed in analyzing the trends of stock prices. Technical analysis is one of the traditional analytical methods that uses historical stock prices and trading volumes to determine the trend of future stock prices. This analysis is based on supply and demand in financial markets and can even be applied to firms with bad financial conditions because this approach only considers historical price data and volumes. Fundamental analysis predicts stock prices by using intrinsic values. The stock values are determined by the financial statements and economic factors of firms. Investors estimate the profits of firms and evaluate whether they are proper. However, this approach cannot reflect other factors that affect stock prices, such as the emotional factor of market participants. Recently, several studies to analyze financial markets with the sentiments of investors, such as blogs, news, and social network services, emerge.

Attempts to forecast stock prices have long been studied and a number of methodologies in various academic fields have been proposed and applied in real markets. Among them, artificial neural network (ANN) has been widely used in classification problems in taking advantage of nonlinearity and has given better performances than other classification methods. Some researches have compared ANN with time series models for predicting time series data. [1] compared the performance of ANN with that of ARIMA model for forecasting commodity prices. The result was that ANN gave a 27% and 56% lower mean squared error than an ARIMA model. [2] have applied ANN and support vector machines (SVM) to predict Istanbul Stock Exhange (ISE) National 100 Index prices. Ten technical indicators were used as inputs and [2] showed maximum 75.74% and 71.52% in ANN and SVM with polynomial kernel respectively. The inputs were only based on the technical factors which use historical index prices and volume data. However, experimental procedures in [2] are not practical to investors in that training and test data was used without considering time series data. Training sets should not be later than test set in analyzing time series data. [3] have employed neural network methods to solve a dynamic and complex problem in the business environment. [3] tested the predictability of stock prices and compared results with multiple discriminant analysis methods. Firms were selected from the Fortune 500 and BusinessWeek and ANN was constructed from inputs of 10 fundamental fators. Stock prices were predicted by the trained ANN into two groups; well-performing firms and poorly-performing firms. The accuracy was 77.5%, which is better than that of multiple discriminant analysis (about 65%). [4] have used various ANN models to predict exchange rates of the NASDAQ index. In [4], the classical ANN model gave better performance than other models and a dynamic architecture of ANN (DAN2) with GARCH models showed the worst performance. [5] used ANNs to predict stock prices on the Tehran Stock Exchange and selected effective accounting factors among 20 factors using a principal component analysis (PCA) method. [6] applied ANNs to the Japanese stock market. To improve the prediction accuracy of ANNs, genetic algorithm and simulated annealing techniques were employed and overcame the local convergence problem of back propagation algorithms. [6] have also proposed 73 input variables including financial indicators and macroeconomic data. The model with the global searching algorithms gave better accuracy than traditional ANNs.

A support vector machine (SVM) is also one of the powerful machine learning methodologies and has been widely used in classification problems in various fields. Classical SVM is a method for linear classification but it can overcome nonlinearity with diverse kernels. There are many researches to predict the trend of financial markets and stocks using SVM. [7] have demonstrated the superiority of SVM compared with ANNs in the trend prediction of the Korea Composite Stock Price Index (KOSPI) by employing 12 technical indicators. [8] forecasted movement directions of NIKKEI 225 index prices using SVM and compared the result with linear discriminant analysis, quadratic discriminant analysis, and Elman backpropagation neural networks. [9] has selected input features from 19 technical indicators by the fractal selection algorithm to train the SVM with radius basis function kernels. [9] also showed that SVMs with fractal feature selection gave the best performance among SVM with other feature selection methods in trend prediction of the Shanghai Stock Exchange Composite Index (SSECI). Similarly [10] have combined a proximal SVM with four feature selection techniques. The performances of four hybrid classifiers were better than the individual proximal SVM and the SVM with random forests was the superiority over all other prediction methods. [11] have combined SVMs with an ARIMA model in statistical models to reflect linear and nonlinear properties. In the hybrid model, residuals obtained from the ARIMA model were modeled by SVMs and can be represented by nonlinear functions.

Opportunities to improve the prediction accuracy of market index prices come to light. First, although some studies have confirmed that improving predictability can be achieved via search frequencies from Google Trends, only a few studies use Google Trends as an input variable. Google Trends developed by Google show the global interests in the subject matter through the frequency of keywords on the Internet. [12] have affirmed that the “early warning signs” of stock market moves can be detected through Google Trends because search frequencies from this web facility result from the interaction of humans with the internet. [13] have also proposed a new measure of investor attentions using search frequencies from Google Trends. They verified that the increase of search frequencies means substantially high stock prices in the next 2 weeks and reversal within the year. They compared Google Trends with the existing proxies of investor attentions. Second, in the case of emerging financial markets, they may be biased toward several large firms and show the co-movement of individual stocks. [14] have confirmed that the cumulative contribution rate of the first two principal components of the Korea Composite Stock Price Index (KOSPI) was approximately 90%. Therefore, the co-movements of individual stocks in the KOSPI may occur, and the variances can be explained by using only two principal components. Given that the Korean financial markets are also weighted toward several large firms, other relatively small firms can be noises to predict market index prices. Most market indices are weighted by the market capitalization of firms and are affected by large firms inevitably. If these scenarios are considered, they may represent improved predictabilities.

This study employs an artificial neural networks (ANN), support vector machines (SVMs) with polynomial kernels, and radial basis function (RBF) kernels to predict the trend of the Korea Stock Price Index 200 (KOSPI 200) prices. We used same input variables with those of [2]’s empirical studies; however, the framework for prediction is reasonably different from theirs. We introduce a framework that is significantly realistic and practical for investors. Moreover, three controversial hypotheses are presented. The first hypothesis aims to compare the general framework with the method and to highlight the nonrealistic parts in [2]. The second hypothesis aims to figure out the effectiveness of Google Trends in prediction. Finally, the third hypothesis aims to determine whether ensemble approaches improve the performance in predicting index prices. Subsequently, we test the hypotheses in our framework and show the results.

## Nonparametric machine learning models

This study employs three nonparametric machine-learning models to analyze the three hypotheses: an ANN, SVMs with polynomial and RBF kernels. These have been widely used in classification problems and have shown good performances.

### Aritificial neural networks

**Fig 1:**
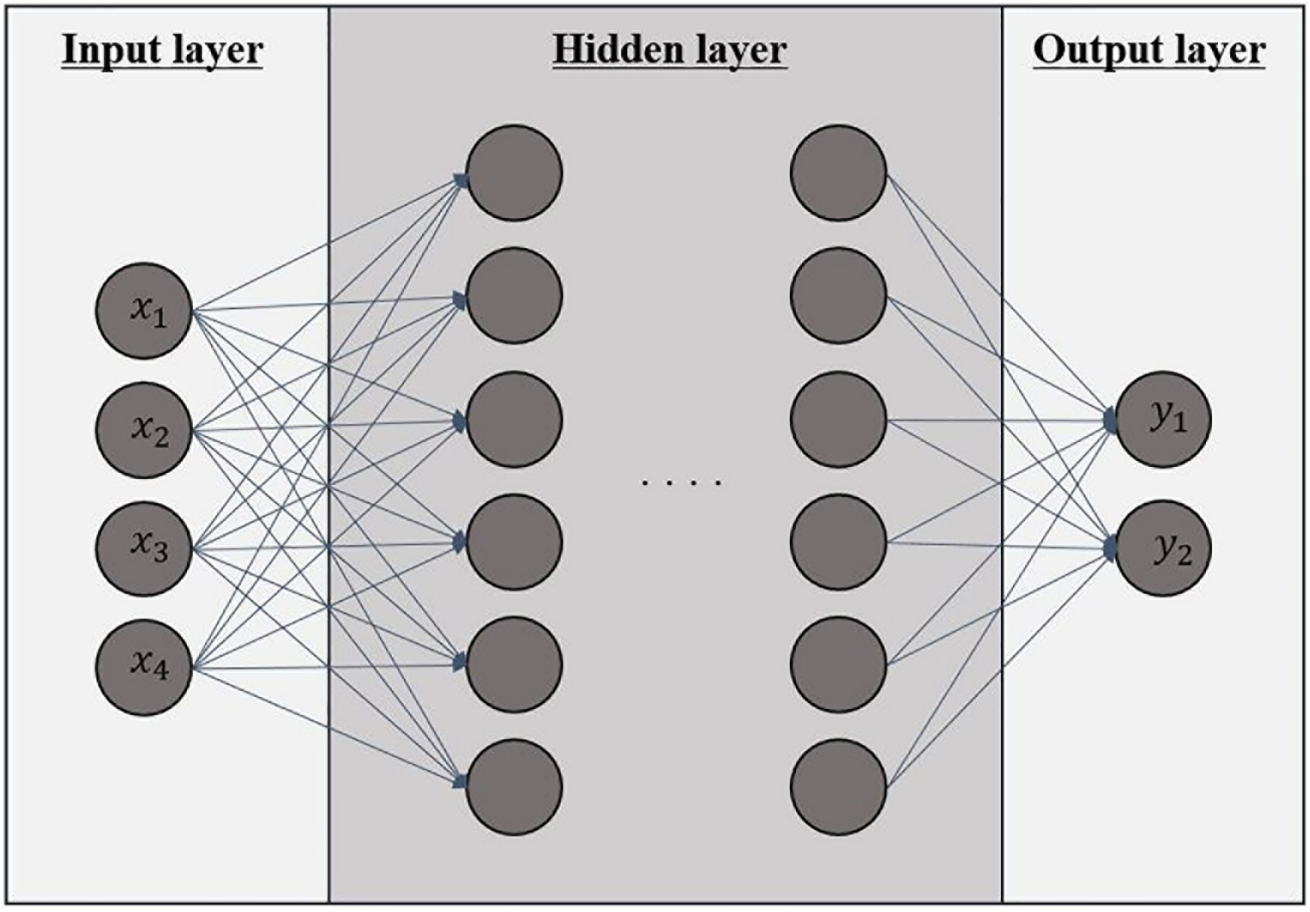
Artificial neural network. There are three layers; an input layer, hidden layers, and an output layer. Inputs are inserted into the input layer, and each node provides an output value via an activation function. The outputs of the input layer are used as inputs to the next hidden layer.

Neural networks developed by [15] are nonparametric nonlinear models which overcome the limitations of linearity. In the networks in Fig 1,

The general way of calculations is as follows:

$$\begin{array}{c} y=f(\sum_{i}w_{i}x_{i}) \end{array}$$ (1)

where *x*_*i*_ is input value from node *i* in the previous layer, *y* is the output value, *f*(⋅) is an activation function, and *w*_*i*_ represents the weight for input *x*_*i*_. Neural networks is a nonlinear regression model because of the activation functions. Neural networks can represent the nonlinear relationship by employing many activation functions such as sigmoid functions, logistic functions, and hyperbolic tangent functions.

The main task of neural networks is to find the optimal weights, *w*_*i*_ in Eq (1). The most widely used method is *back-propagation algorithm* [16]. In this algorithm, the weights are fixed to minimize the loss function between predicted values and true values. The gradients are calculated in backward from the output layer to the input layer. The sum of squared error is usually used as a loss function. Generally, many researchers agree that: the higher the number of hidden layers, the better the performance after optimizing the weights. However it can cause the over-fitting problem and the number of layers should be determined properly according to experiments.

### Support vector machine

**Fig 2:**
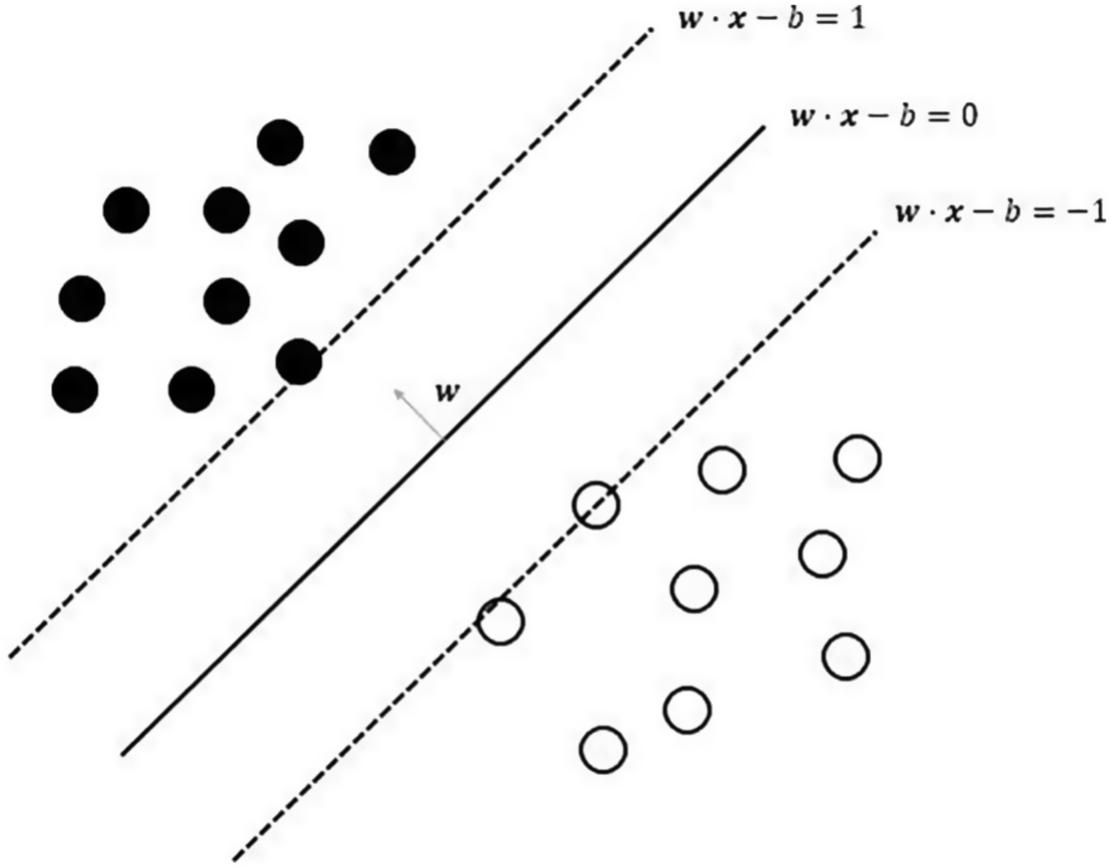
Support vector machine. Given data set of *n* points (**x**_**1**_, *y*_1_), (**x**_**2**_, *y*_2_), …, (**x**_**n**_, *y*_*n*_) where *y*_*i*_ gives -1 or 1 which indicates the class and **x**_**i**_ is a *p*-dimensional vector, we want to classify the points by finding “maximum-margin hyperplane” which divides the data into two groups with at the highest probability. This means that the distance between the hyperplane and the points which are the closest to the hyperplane in each class should be maximized.

A support vector machine (SVM) proposed in [17] is a supervised learning model for the classification and regression. Given the data which belongs to one of two groups, The SVM constructs a non-stochastic binary linear classification model based on the given data and predicts groups of new given data. The classification model can be expressed as a hyperplane that the data is divided by a clear gap, which is as wide as possible. When new data is mapped into the same space with training data, the model can classify the data based on the hyperplane as Fig 2.

Two hyperplanes can be described by Eq (2).

$$\begin{array}{ccc} w\cdot x-b & = & +1\\ w\cdot x-b & = & -1. \end{array}$$ (2)

Because the distance between two hyperplanes is 2/‖**w**‖, we have to minimize ‖**w**‖ to maximize the distance. The hyperplane between halfway of two slashed planes in Fig 2 is called maximum margin hyperplane and the points on the two planes are support vectors.

Geometrically, the points should not be in the region between the planes, so we add the constraint as Eq (3):

$$\begin{array}{ccc} & & w\cdot x-b\geq +1,\;\text{if}\;y_{i}=1\\ & & w\cdot x-b\leq -1,\;\text{if}\;y_{i}=-1. \end{array}$$ (3)

Getting together, the optimization problem is

$$\begin{array}{cc} & \text{Minimize}\;∥w∥\\ \text{subject}\;\text{to} & \\ & y_{i}\left(w\cdot x_{i}-b\right)\geq 1.\;\text{for}\;i=1,\ldots ,n \end{array}$$

By adding a regularization term, we can mitigate the classifier in a linearly inseparable case. The objective function of the equation is expressed as Eq (4),

$$\begin{array}{c} \frac{1}{n}\sum_{i=1}^{n}\text{max}\left(0,1-y_{i}\left(w\cdot x_{i}-b\right)\right)+\lambda {∥w∥}^{2} \end{array}$$ (4)

where the parameter *λ* controls the trade-off between maximizing margin size and being strict in the separation.

SVMs can be applied to nonlinear classification problems by introducing kernel functions, *k*(**x**, **x**′). Polynomial, radial basis function, and hyperbolic tangent kernels are commonly used.

## Data description and procedures

### Data description

This research aims to predict market index prices using the three nonparametric machine-learning models. The inputs are based on 10 technical indicators in [2]. Table 1 presents the inputs.

**Table 1:** Indicators and their formulas.

Name of indicators	Formula
Simple 10-day moving average	$\frac{C_{t}+C_{t-1}+\cdots +C_{t-10}}{10}$
Weighted 10-day moving average	$\frac{\left(n\times C_{t}+\left(n+1\right)\times C_{t-1}+\cdots +C_{10}\right)}{\left(n+(n-1)+\cdots +1\right)}$
Momentum	*C*_*t*_ − *C*_*t*−*n*_
Stochastic K%	$\frac{C_{t}-LL_{t-n}}{HH_{t-n}-LL_{t-n}}\times 100$
Stochastic D%	$\frac{\sum_{i=0}^{n-1}K_{t-i}}{n}$
Relative Strength Index (RSI)	$100-\frac{100}{1+\left(\sum_{i=1}^{n-1}Up_{t-i}/n\right)/\left(\sum_{i=0}^{n-1}Dw_{t-i}/n\right)}$
Moving Average Convergence Divergence (MACD)	MACD(*n*)_*t*−1_ + 2/*n* + 1 × (DIFF_*t*_ − MACD(*n*)_*t*−1_)
Larry William’s R%	$\frac{H_{n}-C_{n}}{H_{n}-L_{n}}\times 100$
Accumulation Distribution Oscillator (A/D)	$\frac{H_{t}-C_{t-1}}{H_{t}-L_{t}}$
Commodity Channel Index (CCI)	$\frac{M_{t}-SM_{t}}{0.015D_{t}}$

*C*_*t*_, *L*_*t*_ and *H*_*t*_ is the closing, low, and high price at time t respectively, *LL*_*t*_ and *HH*_*t*_ is the lowest and highest prices in the last *t* days respectively. DIFF: EMA(12)_*t*_ − EMA(26)_*t*_, Exponential Moving Average(EMA(*k*)_*t*_) = EMA(*k*)_*t*−1_ + (2/(1 + *k*)) × (*C*_*t*_ − EMA(*k*)_*t*−1_) where *k* is time period of *k* day exponential moving average. *M*_*t*_ = (*H*_*t*_ + *L*_*t*_ + *C*_*t*_)/3; $SM_{t}=\left(\sum_{i=1}^{n}M_{t-i+1}\right)/n$, *D*_*t*_ = (∑_*i* = 1_ *n*|*M*_*t*−*i*+1_ − *SM*_*t*_|)/*n*. *Up*_*t*_ is the upward price change and *Dw*_*t*_ is the downward price change at time *t*.

In the present study, data set is the daily closing prices of the KOSPI 200 from 2004 to 2016 freely available from the Korean exchange website: https://global.krx.co.kr/contents/GLB. After normalizing the 10 indicators, we use the indicators as inputs to predict the movement of the index and stock prices. We set the training and prediction data for approximately 6 months and 1 month, respectively. Test data are selected after the training period. We test the machine-learning models using previous data for approximately 6 months and predict the KOSPI200 index for roughly 1 month after the training period. Given that we aim to predict the market index using the technical indicators, we should split the training and test data without overlapping. When analyzing the financial time series data, considering the occurrence time as a variable is important. The data should not be selected randomly. We use this methodology to predict the KOSPI 200 index prices according to several perspectives and compare our results with existing or well-known results by doing hypothesis tests.

### General procedures

**Fig 3:**
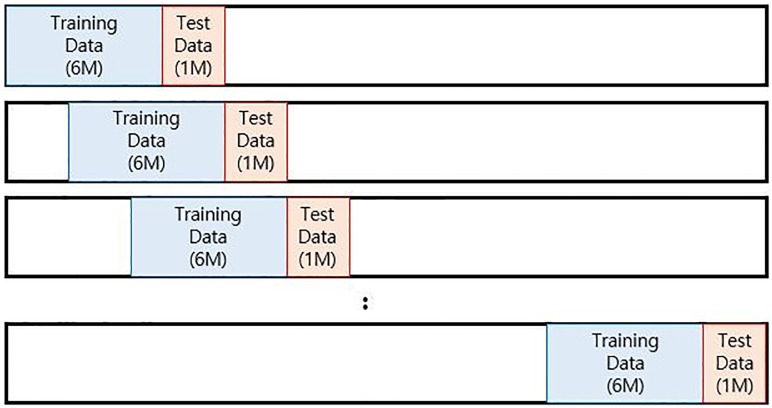
The roll-over strategy. We fix the window for a month and roll over the window to predict next month test data.

However, the framework is entirely different from that of [2]. Therefore, we introduce a roll-over strategy. This strategy has been broadly used to analyze time series data in the fields of economics and management science. This strategy can also be employed in real markets and is practical for investors. Given that the roll-over strategy is based on the past data, investors can use this strategy to predict the index price based on the past prices. Fig 3 presents the aforementioned strategy.

Three parameter sets in each model are selected as the ones that give the best performances in the entire training data. Using estimated parameters, we predict test data and draw accuracy on them.

## Hypothesis tests and results

In this research, three hypotheses are constructed for the analysis of the prediction results of the three machine-learning models. The significant level of the three hypotheses is 0.05, and the data from July 2004 to December 2016 are used as the test data through the roll-over strategy. We assume that the predicted performance of the learned machine is a random variable and each variable follows a normal distribution according to the law of large numbers. Subsequently, we perform a hypothesis test based on the appropriate t-test after performing the equally distributed tests for each variable.

### Regarding on prediction accuracy of machine learning methods

The first hypothesis is based on the results of [2]. In [2], prediction performances in ANNs and SVMs with polynomial and RBF kernel are 75.74%, 71.52% and 62.23%, respectively. Such analysis gives good results in KOSPI 200 index. The results are presented in Table 2. (a, b, c) in ANNs means a parameter set (epochs, momentum constant, number of neurons) and (a, b) in SVMs with polynomial kernel means a parameter set (degree of kernel function, Gamma, regularization parameter). (a, b) in SVMs with RBF means a parameter set (Gamma, regularization parameter).

**Table 2:** Results of the KOSPI200 prediction based on [2] method.

Methods	values			
**ANN**	parameters	(1000, 0.4, 30)	(10000, 0.3, 50)	(1000, 1.0, 40)
	accuracy	79.04%	77.40%	78.91%
**SVM, poly**	parameters	(4, 0.6, 10)	(4, 3.3, 20)	(4, 3.3, 30)
	accuracy	72.98%	71.84%	71.67%
**SVM, RBF**	parameters	(4.9, 10)	(0.1, 20)	(5.0, 30)
	accuracy	72.11%	72.10%	71.86%

This table shows the accuracies of three methods based on [2] framework

To check the robustness, we do experiments using other data; 20-day and 30-day moving average as Table 3. we replace simple 10-day moving average (MA) with two moving average data, respectively. They show better performances than 10-day MA and the ANN gives the best predictability in two experiments, which shows the consistent result with 10-day MA.

**Table 3:** Results of the KOSPI200 prediction based on [2] method with 20-day and 30-day moving average.

MA	Methods	values			
**20-day**	**ANN**	parameters	(3000, 0.1, 20)	(5000, 0.4, 40)	(9000, 1.0, 10)
		accuracy	79.84%	81.34%	77.48%
	**SVM, poly**	parameters	(3, 0.3, 10)	(4, 1.6, 10)	(4, 5.0, 30)
		accuracy	71.63%	70.83%	71.02%
	**SVM, RBF**	parameters	(0.3, 10)	(2.0, 20)	(2.0, 30)
		accuracy	69.35%	72.47%	72.28%
**30-day**	**ANN**	parameters	(1000, 0.1, 10)	(4000, 0.8, 80)	(9000, 1.0, 10)
		accuracy	78.63%	83.01%	78.12%
	**SVM, poly**	parameters	(4, 0.5, 20)	(4, 0.7, 20)	(4, 0.6, 30)
		accuracy	69.34%	69.16%	69.47%
	**SVM, RBF**	parameters	(1.2, 10)	(0.6, 20)	(0.2, 30)
		accuracy	70.41%	69.99%	70.60%

This table shows the accuracies of three methods based on [2] framework with 20-day and 30-day moving average.

The procedures in [2] are inappropriate to predict the market index. The data set for parameter estimations consists of 20% of each increasing and decreasing direction equally, and the test data set for the prediction consists of 50% in the same principle. In this procedure, the data set for parameter estimations can also be selected to predict the test data. This method is not realistic in the investors’ view because a real practical application cannot be used the data for parameter setting or training to predict the direction of markets investors want to know. Selecting training and prediction data sets in similar numbers of increasing and decreasing directions are not realistic and useless to apply in actual trading strategies. Therefore, we set the training data for approximately six months and then the prediction data for roughly one month according to our general procedure. Based on these two methods, we test a hypothesis: the prediction performances of each method are similar. This hypothesis is intended to determine whether the high accuracy of the machine-learning method previously reported is independent of the procedures that deal with the data.

Prior to the hypothesis testing, the Anderson-Darling test was performed to samples from in [2] frameworks and the two-sample F-test for equal variances were performed. Given that the Anderson-Darling test did not reject the null hypothesis, the assumption that the sample follows a normal distribution can be allowed. Two independent sample t-tests were performed because the equal variance test rejects the null hypothesis. The null hypothesis and the alternative hypothesis are as follows. The mean value of prediction based on [2] and our framework are *μ*_*kara*_ and *μ*_*nonoverlap*_, respectively.

$$\begin{array}{cc} H_{0}:\; & \mu_{kara}=\mu_{nonoverlap}\\ H_{1}:\; & \mu_{kara}\neq \mu_{nonoverlap} \end{array}$$

Given the constraints of the model framework, we have 13 data sets for [2] framework and 146 data sets for the non-overlapping framework. We generate a power curve to visualize how the sample size affects the test power. Table 4 shows that the p-value for the null hypothesis is significantly low, which means that, even with very few samples, the average values of the two frames are different. In the first hypothesis test, the result in [2] is different from our result with general procedure that is realistic and practical for actual investors. All models reject the null hypothesis that the mean value of prediction based on [2] is equal to the mean value of prediction based on our general framework.

**Table 4:** Results of the first hypothesis test.

	ANN	SVM, poly	SVM, RBF
**p-value**	2.00 × 10^−322^	9.53 × 10^−199^	1.16 × 10^−189^
**reject/fail to reject *H*_0_**	reject	reject	reject

This table shows results of the first hypothesis test. All methods reject the null hypothesis.

We check robustness about the first hypothesis with 20-day and 30-day MA in Table 5. We replace 10-day MA with 20-day and 30-day MA, respectively. The results also show that all of null hypothesis are rejected, which shows consistency of results.

**Table 5:** Results of the first hypothesis test with 20-day and 30-day MA.

		ANN	SVM, poly	SVM, RBF
**20-day**	**p-value**	6.48 × 10^−52^	2.43 × 10^−27^	3.58 × 10^−14^
	**reject/fail to reject *H*_0_**	reject	reject	reject
**30-day**	**p-value**	9.72 × 10^−42^	3.10 × 10^−14^	2.07 × 10^−17^
	**reject/fail to reject *H*_0_**	reject	reject	reject

This table shows results of the first hypothesis test with 20-day and 30-day MA for robustness. All methods reject the null hypothesis consistently.

**Fig 4:**
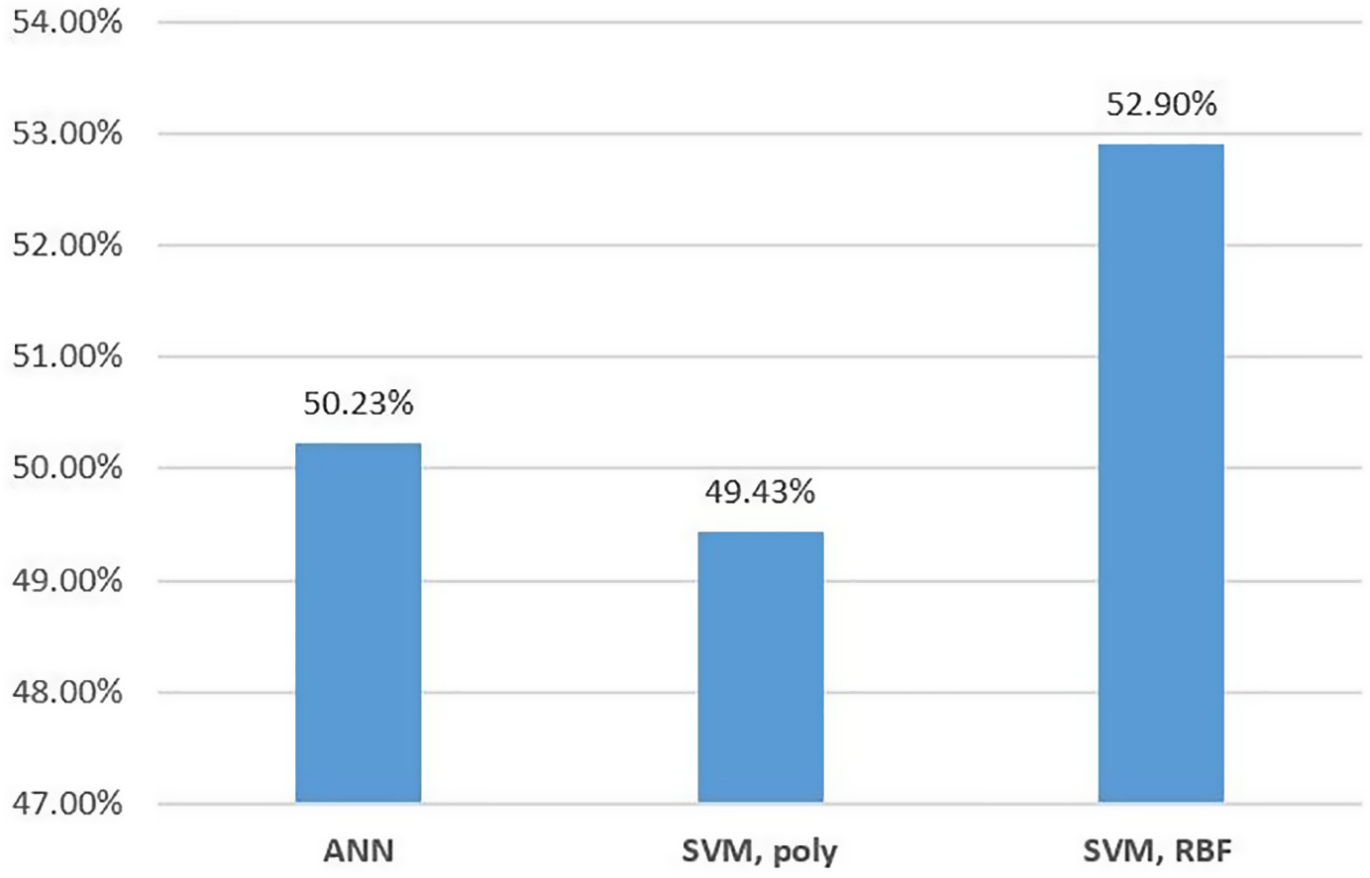
Accuracies of prediction of KOSPI 200.

Fig 4 depicts the prediction accuracy of the KOSPI 200 index prices in three machine-learning models. Support vector machines with RBF give the best performance in predicting the KOSPI 200 index prices. However, the accuracy of the three models is almost 50%. Only two directions should be predicted. Machine-learning methods with general procedures do not give good performances in predicting the trends of market index prices. In other words, the models with the 10 indicators do not have high predictabilities based on the past training data.

### Regarding on predicting trends of the market indexes with Google Trends

Various analytical methods have been developed using large data that evokes attention recently. Google Trends that shows search frequencies from Google is one of investors’ sentiment indicators because the records are made from market participants or potentials. To verify the effect of Google Trends, we add the search frequency of the KOSPI 200 index as an input in our framework. This approach allows us to determine whether the prediction with Google Trends provides better performances compared with the prediction without this web search frequency.

$$\begin{array}{cc} H_{0}:\; & \mu_{w/google}=\mu_{w/ogoogle}\\ H_{1}:\; & \mu_{w/google}\neq \mu_{w/ogoogle} \end{array}$$

**Fig 5:**
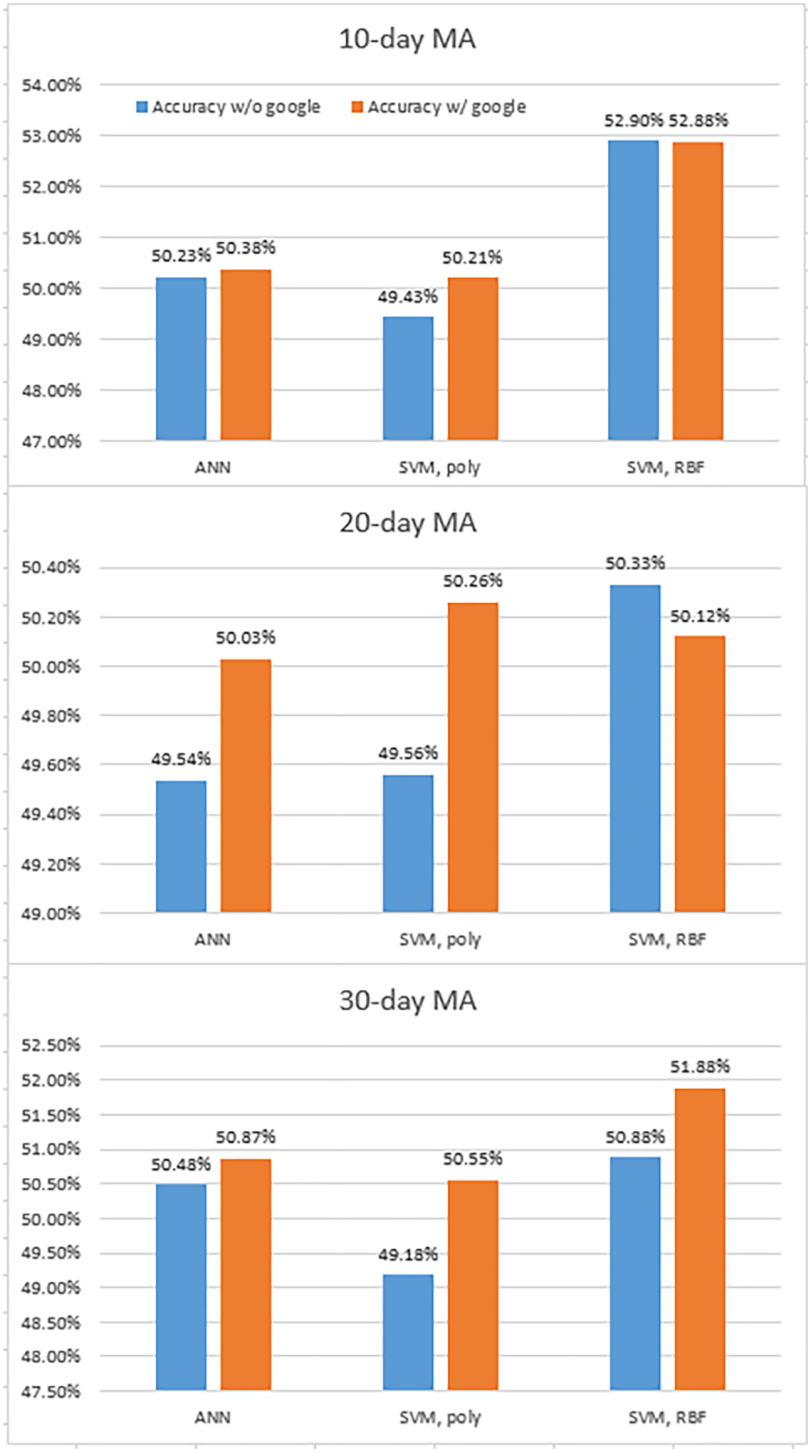
Accuracies of prediction of with and without Google Trend.

T-test was performed after adopting the equal variance assumption by preferentially performing the equally distributed tests as likely in the hypothesis test 1. In addition, search frequencies from Google Trends are added as an input variable. Fig 5 and Table 6 show the results of prediction and t-test. While the ANN and SVM with polynomial kernels might have better performances with Google Trends compared with without it, the SVM with RBF kernels might give similar performances. However, it is difficult to determine the effectiveness of Google Trends as shown in Fig 5. All the models fail to reject the null hypothesis. Moreover, the second hypothesis test confirms that Google Trends is an ineffective factor in predicting the KOSPI 200 index prices in the current framework. Unlike previous studies on the impact of Google Trends on investment decisions, this study validates that using the Google Trends information as an input may not always be effective for all machine-learning models. This scenario may be because Google Trends is not suitable for applying to the KOSPI 200 index data or is not appropriate for the ANN or SVMs, thereby suggesting further research in this area.

**Table 6:** Results of the second hypothesis test.

	ANN	SVM, poly	SVM, RBF
**p-value**	0.8572	0.5452	0.9256
**reject/fail to reject *H*_0_**	fail to reject	fail to reject	fail to reject

This table shows results of the second hypothesis test. All methods fail to reject the null hypothesis.

The second hypothesis with 20-day and 30-day MA also shows consistent results in Table 7. All models fail to reject the null hypothesis, which means Google Trends can be an ineffective factor in predicting the index prices.

**Table 7:** Results of the second hypothesis test with 20-day and 30-day MA.

		ANN	SVM, poly	SVM, RBF
**20-day**	**p-value**	0.7081	0.7343	0.8532
	**reject/fail to reject *H*_0_**	fail to reject	fail to reject	fail to reject
**30-day**	**p-value**	0.6743	0.8652	0.8371
	**reject/fail to reject *H*_0_**	fail to reject	fail to reject	fail to reject

This table shows results of the second hypothesis test with 20-day and 30-day MA for robustness. All methods fail to reject the null hypothesis consistently.

### Regarding on Korean financial market prediction through ensemble techniques

Market index consists of many companies according to its characteristics. The KOSPI 200 index is a market capitalization index that consists of 200 representative companies in South Korea. The Korean market represents a biased trend toward some large companies. Other companies, except for large ones, are relatively less likely to explain the trend of market index prices. Generally, machine-learning methods will probably improve their performances through ensemble techniques. Therefore, we attempt to compare the predictive performance of the whole market index with those of the ensemble approaches with major companies in the index. We select 10 representative companies with large capitalization among the 200 companies in the KOSPI 200 index. The same framework is applied to predict the trends of each company with the 10 technical indicators.

$$\begin{array}{cc} H_{0}:\; & \mu_{ensemblewithTOP10}=\mu_{KOSPI}\\ H_{1}:\; & \mu_{ensemblewithTOP10}\neq \mu_{KOSPI} \end{array}$$

The third hypothesis concentrates on the prediction of market index prices’ direction using the ensemble methods with the results of each individual major company in the market index. We select 10 representative companies with market capitalization in the KOSPI 200 index. If ensemble prediction performances with individual companies are better compared with employing the whole index, then the ensemble methods are effective to forecast the trend of market index prices.

**Fig 6:**
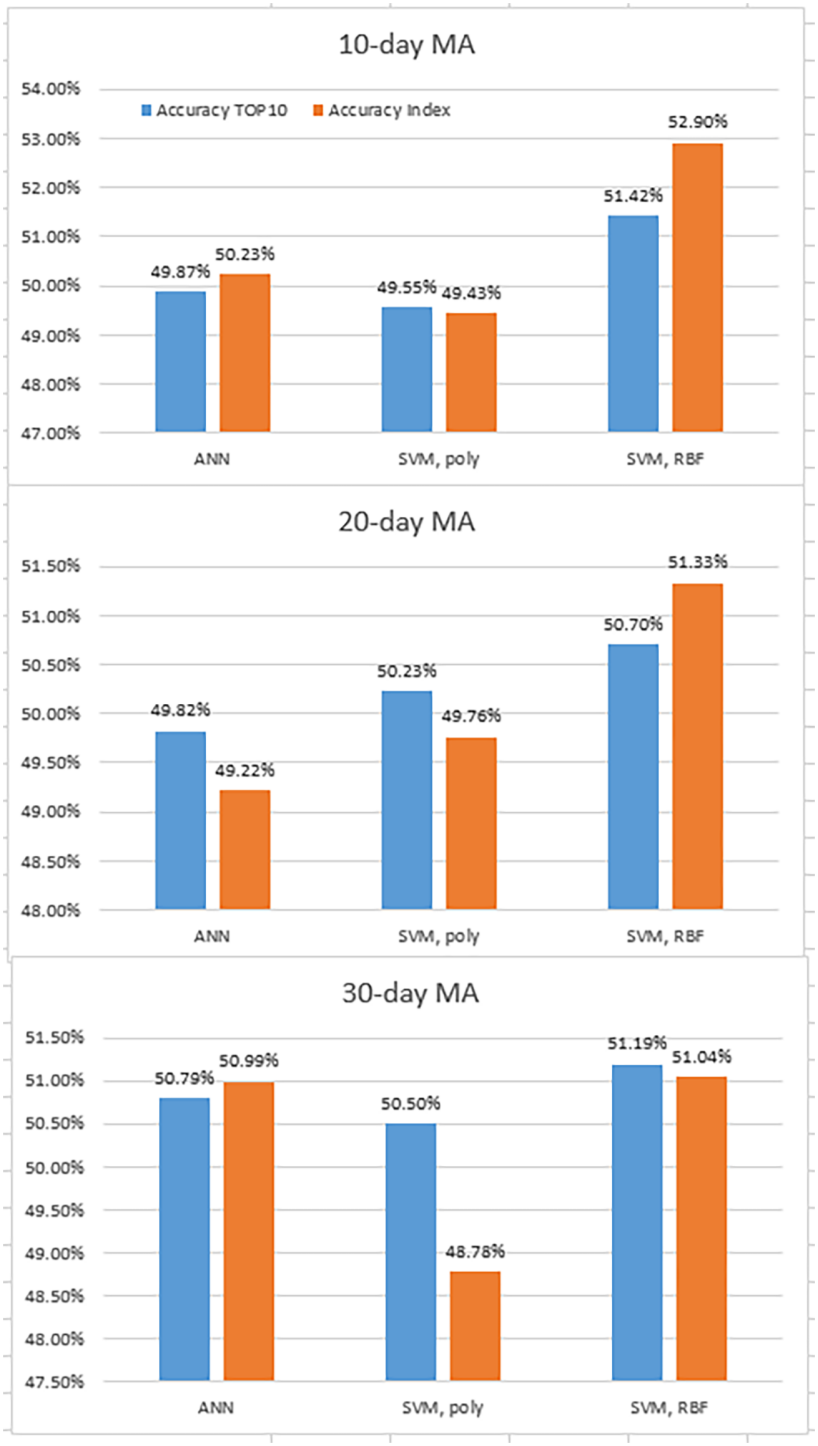
Accuracies of prediction using individual companies and KOSPI 200.

In this empirical study, we predict the stock direction of individual companies with 10 indicators for each company. If the weighted average of directions based on market capitalization is larger than 0.5, then the market index is expected to increase. Fig 6 shows the result. While the SVM with RBF kernels provide significant performances when predicting the index using the 10 representative companies, the ANN and SVM with polynomial kernels show similar accuracies between two predictions.

Table 8 shows the result of the third hypothesis test. All the models fail to reject the null hypothesis that the prediction that employs the 10 representative companies provides better performances compared with employing only the whole index. Therefore, the ensemble methodology has no visible effect on the directionality of the market index. The hypothesis that investors can predict the Korean financial markets by solely looking at large companies is, hence, inaccurate. Accordingly, the performance of the ensemble techniques depends on the specific machine-learning methodology and the data.

**Table 8:** Result of the second hypothesis test.

	ANN	SVM, poly	SVM, RBF
**p-value**	0.9824	0.5130	0.7963
**reject/fail to reject *H*_0_**	fail to reject	fail to reject	fail to reject

This table shows result of the third hypothesis test. All methods fail to reject the null hypothesis.

We check robustness in this experiment. In Table 9, the models with 20-day and 30-day MA give consistent results to the experiment with 10-day MA, which satisfies robustness of the models.

**Table 9:** Results of the third hypothesis test with 20-day and 30-day MA.

		ANN	SVM, poly	SVM, RBF
**20-day**	**p-value**	0.6393	0.8252	0.7376
	**reject/fail to reject *H*_0_**	fail to reject	fail to reject	fail to reject
**30-day**	**p-value**	0.5604	0.1682	0.8819
	**reject/fail to reject *H*_0_**	fail to reject	fail to reject	fail to reject

This table shows results of the third hypothesis test with 20-day and 30-day MA for robustness. All methods fail to reject the null hypothesis consistently.

## Conclusion

Predicting stock prices has been a major issue to investors and researchers. Many methodologies from various academic fields have been introduced for prediction, and some methods have been used in real financial markets for trading strategies. Recently, researchers have analyzed the trends of stock prices using machine-learning models, and they have shown considerable performances by using meaningful input factors.

In this research, we elucidate disassociation points with real trading environments in analyzing the time series data and in leveraging realistic and practical methods for actual investors. To predict the movement of the KOSPI 200 index prices, we use the three nonparametric machine-learning models: ANN, SVMs with polynomial kernels, and RBF kernels. Applying the methods to various circumstances, we deal with controversial issues by testing three hypotheses. In the first hypothesis, we prove that the prediction of the KOSPI 200 using the 10 technical indicators introduced by [2] does not give a good performance in plausible framework for market investments. In addition, the result of [2] is not consistent with the plausible framework result that predicts future data only based on the past training data. In the second hypothesis, we affirm that Google Trends may be an inadequate input factor in predicting the KOSPI 200 index prices. Accordingly, the second hypothesis test suggests that the suitability of Google Trends for the analysis should be evaluated before other uses in applications. In the third hypothesis, we confirm that the ensemble study results with the 10 selected large companies in the KOSPI 200 index are similar to the whole index prediction results unlike the prediction studies based on the machine-learning techniques.

This study confirmed the instability and large variability of machine-learning methods on market forecasts, which is mentioned in [18] and some other researches, through hypothesis testing using the case of the KOSPI 200 index market. It implies possibilities to be extended on some points. Several limitations of machine-learning methods for KOSPI index market are pointed out in this study. Thus, each limitation can be analyzed in detail and can ameliorate the prediction accuracy of KOSPI 200 index markets. [19] have found Google Trends may help in predicting the near-term values of economic indicators. Google Trends may be effective in predicting the future of the KOSPI index market in a shorter interval than a month. In the case of ensembles using machine learning, there is a possibility of improving the index market prediction results by using related techniques such as bagging and boosting.

## Supporting information

S1 File Raw trading data for KOSPI 200 index. The following data includes the trading dates, open prices, high prices, low prices, close prices, trading volumes and Google Trends of KOSPI 200 Index from 2004 to 2016.
(XLSX)

S2 File Raw trading data for 10 major companies in KOSPI 200 index. The following data includes the trading dates, open prices, high prices, low prices, close prices, trading volumes and market capitalization of major 10 companies in KOSPI 200 Index from 2004 to 2016.
(XLSX)

S3 File Matlab code of experiments in hypothesis 1. The following code includes experiments in [2] in Matlab. It includes parameter setting, training and test procedure of ANN and SVM with two kernels and robustness test.
(M) Click here for additional data file.

S4 File Matlab code of experiments with Google Trends. The following code includes experiments in hypothesis 1 and 2. The experiment without Google Trends is used in hypothesis 1 as a non-overlap method and hypothesis 2 as a prediction without Google Trends. The experiment with Google Trends is used in hypothesis 2 to be compared to that without Google Trends and robustness test.
(M) Click here for additional data file.

S5 File Matlab code of experiments in hypothesis 3. The following code includes experiments in hypothesis 3. The experiment with TOP 10 companies in KOSPI 200 is done to be compared to that with only using KOSPI 200 index prices and robustness test.
(M) Click here for additional data file.

S6 File Matlab code of kernel functions for SVM methods. The following code includes kernel functions to carry out experiments related with the SVM method. This function code is employed for all hypothesis testing experiments.
(M) Click here for additional data file.

## References

[1] Kohzadi N, Boyd MS, Kermanshahi B, Kaastra I. A comparison of artificial neural network and time series models for forecasting commodity prices. Neurocomputing. 1996;10(2):169–181. doi: 10.1016/0925-2312(95)00020-8

[2] Kara Y, Boyacioglu MA, Baykan ÖK. Predicting direction of stock price index movement using artificial neural networks and support vector machines: The sample of the Istanbul Stock Exchange. Expert Syst Appl. 2011;38(5):5311–5319. doi: 10.1016/j.eswa.2010.10.027

[3] Yoon Y, Swales G. Predicting stock price performance: A neural network approach. In: System Sciences, 1991. Proc Annu Hawaii Int Conf Syst Sci. vol. 4. IEEE; 1991. p. 156–162.

[4] Guresen E, Kayakutlu G, Daim TU. Using artificial neural network models in stock market index prediction. Expert Syst Appl. 2011;38(8):10389–10397. doi: 10.1016/j.eswa.2011.02.068

[5] Zahedi J, Rounaghi MM. Application of artificial neural network models and principal component analysis method in predicting stock prices on Tehran Stock Exchange. Phys A. 2015;438:178–187. doi: 10.1016/j.physa.2015.06.033

[6] Qiu M, Song Y, Akagi F. Application of artificial neural network for the prediction of stock market returns: The case of the Japanese stock market. Chaos Solitons Fractals. 2016;85:1–7. doi: 10.1016/j.chaos.2016.01.004

[7] Kim Kj. Financial time series forecasting using support vector machines. Neurocomputing. 2003;55(1):307–319. doi: 10.1016/S0925-2312(03)00372-2

[8] Huang W, Nakamori Y, Wang SY. Forecasting stock market movement direction with support vector machine. Comput Oper Res. 2005;32(10):2513–2522. doi: 10.1016/j.cor.2004.03.016

[9] Ni LP, Ni ZW, Gao YZ. Stock trend prediction based on fractal feature selection and support vector machine. Expert Syst Appl. 2011;38(5):5569–5576. doi: 10.1016/j.eswa.2010.10.079

[10] Kumar D, Meghwani SS, Thakur M. Proximal support vector machine based hybrid prediction models for trend forecasting in financial markets. J Comput Sci. 2016;17:1–13. doi: 10.1016/j.jocs.2016.07.006

[11] Pai PF, Lin CS. A hybrid ARIMA and support vector machines model in stock price forecasting. Omega. 2005;33(6):497–505. doi: 10.1016/j.omega.2004.07.024

[12] Preis T, Moat HS, Stanley HE. Quantifying trading behavior in financial markets using Google Trends. Sci Rep. 2013;3:1684 doi: 10.1038/srep01684 23619126 10.1038/srep01684 PMC3635219

[13] Da Z, Engelberg J, Gao P. In search of attention. J Finance. 2011;66(5):1461–1499. doi: 10.1111/j.1540-6261.2011.01679.x

[14] Wang Y. Stock price direction prediction by directly using prices data: an empirical study on the KOSPI and HSI. Int J Bus In Data Min. 2014;9(2):145–160.

[15] Rosenblatt F. Principles of neurodynamics. perceptrons and the theory of brain mechanisms. DTIC Document; 1961.

[16] Rumelhart DE, Hinton GE, Williams RJ. Learning internal representations by error propagation. DTIC Document; 1985.

[17] Vapnik V. The nature of statistical learning theory. Springer science & business media; 2013.

[18] Preethi G, Santhi B. Stock market forecasting techniques: a survey. J Theor Appl Inf Tech. 2013; 46(1).

[19] Choi H, Varian H. Predicting the present with Google Trends. Econ Re. 2012;88(s1):2–9. doi: 10.1111/j.1475-4932.2012.00809.x

